# Risk factors of opioid use associated with an enhanced-recovery programme after total knee arthroplasty

**DOI:** 10.1186/s12891-021-04937-8

**Published:** 2021-12-20

**Authors:** Guorui Cao, Shiqi Xiang, Minglu Yang, Songtao Quan, Junna Yao, Litao Cai, Wei Feng, Xiuli Yang, Hong Xu, Zeyu Huang, Shaoyun Zhang, Chen Yue, Honglue Tan, Fuxing Pei

**Affiliations:** 1 Department of Knee Injury, Luoyang Orthopedic Hospital of Henan Province, Orthopedic Hospital of Henan Province, Luoyang, Henan Province People’s Republic of China; 2 Department of Orthopedics, The Second Xiangya Hospital of Central South University, Changsha, Hunan Province People’s Republic of China; 3 Department of Orthopaedic surgery, West China Hospital, Sichuan University, 37# Guoxue Road, Chengdu, Sichuan Province 610041 People’s Republic of China; 4 Department of Orthopedics, The Third Hospital of Mianyang, Sichuan Mental Health Center, Mianyang, Sichuan Province People’s Republic of China

**Keywords:** Opioid, Risk factors, Total knee arthroplasty, Length of stay

## Abstract

**Background:**

Characterizing the impacts of postoperative opioid use on total knee arthroplasty (TKA) patients may help optimize the pain management after TKA. The aim of the study is to examine the prevalence and risk factors for opioid use with an enhanced-recovery programme after primary TKA.

**Methods:**

We identified 361 patients undergoing TKA, and separated those on the basis of whether to receive opioid use after surgery. Themultivariate logistic regression model was used to identify independent risk factors for opioid use after primary TKA. Length of stay (LOS) and postoperative complications were also recorded and compared.

**Results:**

The prevalence of opioid use after primary TKA was 23.0%. The significant risk factor was the longer operative time (OR [odds ratio] = 1.017, 95% CI [confidence interval] = 1.001 to 1.032, *p* = 0.034) and the protective factor was the utilization of tranexamic acid(OR= 0.355, 95% CI = 0.161 to 0.780, *p* = 0.010). In addition, the LOS was longer in opioid group (*p* < 0.05).

**Conclusion:**

Considering the adverse health effects of opioid use, strategies need to be developed to prevent persistent opioid use after TKA. Reducing operative time and the application of tranexamic acid could lower the risk of opioid use with an enhanced-recovery programme after primary TKA.

## Introduction

Total knee arthroplasty (TKA) is a common procedure to treat advanced osteoarthritis of the knee and the number of TKA is growing annually [1, 2]. Due to the emergence of the significant pain in the early postoperative period following TKA,the pain management has been considered a challenging issue [3, 4]. Appropriate pain management consist of patient education, physical therapy and medications, including acetaminophen, non-steroidal anti-inflammatory drugs (NSAIDs) and opioid [5].

Opioid has been proposed as one of the effective methods to treat choric pain after TKA, many patients in America even receive opioid for more than 3 months to control pain postoperatively [6]. However, it is reported that postoperative opioid utilization could also cause a series of side effects, such as delayed rehabilitation, higher revision risk, opioid dependency and withdrawal [7–9]. , Despite it, as the strongest analgesic agent for treating the postsurgical patients, opioid still be considered as the important remedial pain management drug for TK A[4]..

Previous researchers used opioid as a routine means of analgesia and the postoperative utilization rate was approximately 90%. The risk factors for prolonged opioid use and opioid-related adverse drug events after TKA were extensively studied in previous studies [10–13]. With the development of enhanced recovery following joint surgery, preemptive analgesia, multimodal analgesia as well as the application of tranexamic acid (TXA) and dexamethasone relieved pain intensity after TKA. Considering the side effects of opioid, opioid has been only taken as a remedy when postoperative pain control was poor in our clinic center. Since there is no perfect pain management protocol, it is quite necessary to investigate the relationship between TKA and the usage of opioid. The prevalence and related factors associated with whether or not use opioid after primary TKA are still poorly studied. Therefore, we perform this retrospective cohort study to investigate the risk factors associated with postoperative opioid use following primary TKA.

## Materials and methods

### Study design and study sample

We conducted a single-center retrospective cohort study and enrolled patients undergoing primary TKA owing to osteoarthritis and rheumatoid arthritis from June 2016 to January 2019. The study was approved by the hospital’s Institutional Review Board. This study was reported using the Strengthening the Reporting of Observational Studies in Epidemiology guidelines [14].

The patients were excluded with any of the following situations: cardiovascular problems (myocardial infarction, cardiac insufficiency, and other serious heart diseases evaluated by physicians), history of chronic musculoskeletal pain or other types of chronic pain, history of cancer, and incomplete medical records. Overall, 361 patients were included in this study. The enrolled patients were divided into the opioid group or non-opioid group according to whether patients received opioid drugs to control pain after primary TKA.

### Surgical Technique and Perioperative protocol

All the surgeries were performed under general anesthesia by the same senior surgeon, through a midline skin incision, medial parapatellar approach, and a measured resection technique. The prosthesis was cemented posterior-stabilized prosthetic design with patellar resurfacing. 200 mg ropivacaine diluted in 100 mL of normal saline solution was injected periarticularly when the deep fascia was sutured. No tourniquet, nerve block, intravenous patient-controlled analgesia or blood salvage system were used. The operative time and intraoperative blood loss were recorded carefully.

All patients received enhanced recovery protocols, including blood management, sleep management and so on [15, 16]. The detailed strategy was showed in Table 1. The physical prophylaxis and chemoprophylaxis were applied to prevent DVT and PE based on our previous study [3]. The TXA (20 mg/kg) was administered intravenously 5-10 minutes before skin incision, along with 1 g intravenous TXA (diluted in 100 mL of normal saline solution) at 3, 6, 12, and 24 hours postoperatively.

**Table 1:** The strategy of enhanced recovery after primary TKA

	Detailed measures
Blood Management	Correcting perioperative anemia, including iron supplement, erythropoietin and transfusion. Multiple TXA application: 5-10 min preoperatively and 3, 6, 12, and 24 hour postoperatively. Operation: operating carefully and cutting layer by layer.
Pain Management	Preoperative education Preemptive analgesia: oral Celecoxib preoperatively more than 1 week. Multimodal analgesia: oral NSAIDs postoperatively 2-4 weeks and 200 mg ropivacaine local infiltration around articular cavity intraoperatively. Remedy: Opioid.
Nutrition Management	Preoperative early-stage feeding:receiving carbohydrate powder 2 hour and protein powder 6 hour dissolved in normal saline before the induction of anesthesia. Postoperative diet starting 2–4 hours after surgery.

Routinely, all patients received oral Celecoxib (200 mg twice a day, Celebrex; Pfizer, New York) preoperatively and oral enteric-coated diclofenac sodium (50 mg twice a day, Voltaren; Novartis, Basel, Switzerland) postoperatively for 2-4 weeks after surgery. 200 mg ropivacaine dissolved in 60 mL normal saline was infiltrated around articular cavity when the prosthesis was placed. Cold pack and intermittent foot slope pump systems were given to all patients for 12-24 hours postoperatively. The Visual Analogue Scale (VAS) score was measured and recorded preoperatively and postoperative every day (Twice a day, 8 a.m. and 6 p.m.) in motion and at rest by surgeon and nurses before discharging hospital. Using opioid drug (OxyContin or oxycodone hydrochloride sustained release tablets) once or more after surgery was defined as opioid use. The opioid was applied when the VAS score was ≥ 5 in motion or ≥ 3 in rest. The dosage was 10 mg twice a day, increasing gradually when pain control was poor (VAS score was ≥ 5 in motion or ≥ 3 in rest) and the maximum of dosage was 100 mg. The surgeon decided when to prescribe opioids according to pain control situation.

### Outcome Measurements

The number of patients who received opioid prescriptions after TKA was the outcome of interest. Perioperative variables considered as potential risk factors, including age, gender, diagnosis, body mass index, medical comorbidities and laboratory markers were collected. In addition, the American Society of Anesthesiologists physical status classification, the application of TXA and drainage, operative time, intraoperative blood loss, length of stay (LOS) after surgery, and postoperative complications were carefully recorded.

### Statistical analyses

Descriptive and univariate analyses were conducted to compare the characteristics in patients with or without opioid use after primary TKA. A multivariable logistic regression model was used to examine independent risk factor for postoperative opioid use in TKA. Factors associated with postoperative opioid use with *p* < 0.10 were selected for inclusion in the multivariable model. Collinearity was checked using tolerance values (p >0.10). All analyses were performed using SPSS version 22.0(SPSS Inc. USA). A *p* value of <0.05 was considered statistically significant.

## Results

Totally, 361 patients were enrolled in the study. Among them, 83 patients received opioid drugs to control pain after primary TKA. The prevalence of opioid use was 23.0%. The results of comparison between the opioid and non-opioid group were shown in Table 2. The variables that were selected for the logistic regression model after univariate analyses were smoking, TXA use, more operative time and intraoperative blood loss (*p* < 0.10).

**Table 2:** The comparison of patients with or without opioid use following primary TKA

Baseline Characteristic	Opioid group, N =83	Non-opioid group, N =278	*p*
**Demographic characteristics**			
Age($\overline{X}$±S)	66.1±8.9	66.0±7.7	0.897
Sex (male/female)	19/64	45/233	0.160
BMI($\overline{X}$±S, kg/*m*^2^)	25.6±3.5	25.4±3.6	0.880
Diagnosis (RA/OA)	5/78	8/270	0.177
Preoperative HSS	48.7±8.9	49.3±8.2	0.582
Preoperative VAS	4.9±0.7	4.9±0.8	0.578
Preoperative varus (N, %)	45 (54.2%)	149 (53.6%)	0.921
Preoperative valgus (N, %)	8 (9.6%)	30 (10.8%)	0.764
**Comorbidities (N, %)**			
Smoking	10 (12.0%)	17 (6.1%)	0.071
Alcohol	5 (6.0%)	16 (5.8%)	0.927
Hypertension	48 (57.8%)	167 (73.3%)	0.715
Diabetes	10 (12.0%)	48 (21.1%)	0.256
Coronary heart disease	10 (12.0%)	20 (7.2%)	0.160
Preoperative anemia	19 (22.9%)	62 (22.3%)	0.910
COPD	8 (9.6%)	22 (7.9%)	0.617
**Preoperative laboratories**			
HB (g/L)	131.3±13.4	130.7±12.9	0.698
Hct	39.7±3.7	39.9±3.6	0.746
Albumin (g/L)	42.8±3.2	43.4±3.2	0.191
ESR (mm/h)	24.0±16.3	27.0±18.0	0.259
CRP (mg/L)	3.5±4.28	3.6±3.0	0.748
IL-6 (mg/L)	4.3±2.9	4.2±4.1	0.771
Platelet (*10^9^/L)	183.5±68.6	187.6±59.6	0.635
PT (sec)	11.4±0.7	11.3±1.0	0.670
APTT (sec)	27.7±3.1	27.5±3.3	0.751
**Operative variables**			
ASA class (N, %)			0.789
1-2	70 (84.3%)	231 (83.1%)	
≥3	13 (15.7%)	47 (16.9%)	
Drainage use (N, %)	22 (26.5%)	88 (31.7%)	0.371
TXA use (N, %)	71 (85.5%)	259 (93.2%)	0.030*
Dexamethasone use (N, %)	65 (78.3%)	234 (84.2%)	0.214
Operative time (min)	80.4±16.2	76.7±15.0	0.056
Intraoperative blood loss (mL)	127.4±33.0	104.6±29.2	<0.001*

BMI: body mass index; RA: rheumatoid arthritis; OA: osteoarthritis; HSS: hospital for special surgery knee score; VAS: visual analogue scale; COPD: chronic obstructive pulmonary disease; HB: hemoglobin; Hct: hematocrit; ESR: erythrocyte sedimentation rate; CRP: C reaction protein; IL-6: interleukin-6; PT: prothrombin time; APTT: activated partial thrombin time; ASA, American Society of Anesthesiologists; TXA: Tranexamic acid.

*p* value calculated using independent t-test, Pearson chi-square test or Fisher exact test.

*Significant difference

Risk factors independently associated with opioid use was the longer operative time (OR [odds ratio] = 1.017, 95% CI [confidence interval]) = 1.001 to 1.032, *p* = 0.034). Moreover, the administration of TXA was a protective factor for opioid use after TKA (OR = 0.355, 95% CI = 0.161 to 0.780, *p* = 0.010) (Table 3).

**Table 3:** Logistic regression models for opioid use following primary TKA

Parameter	Odds Ratio	95% Confidence Interval	*p*
Smoking	2.274	0.988-5.234	0.054
TXA use	0.355	0.161-0.780	0.010*
Operative time	1.017	1.001-1.032	0.034*

TXA: Tranexamic acid.

*Significant difference

The LOS and related complications were summarized in Table 4. The LOS in opioid group was longer than that in non-opioid group (3.8 ± 1.2 vs 3.5 ± 0.9, *p* = 0.36). In addition, the incidence of nausea and vomiting was higher in opioid group (25.3% vs 11.2%, *p* = 0.001). The incidence of other complications, including deep venous thrombosis, pulmonary embolism, wound complications and so on, did not reach statistical difference between the two groups (*p* > 0.05)

**Table 4:** LOS, expenses and Complications

Variables	Opioids group, N =83	Non-opioids group, N =278	*p*
LOS	3.8±1.2	3.5±0.9	0.036*
Transfusion	1 (1.2%)	1 (0.4%)	1.000
Death	0	0	-
DVT	0	4 (1.4%)	0.489
PE	0	0	-
Nausea and vomiting	21 (25.3%)	31 (11.2%)	0.001*
Cardiac infarction	1 (1.2%)	0	0.230
Stroke	0	0	-
Acute renal failure	0	0	-
Wound complications	16 (19.3%)	37 (13.3%)	0.178
Readmission	2 (2.4%)	2 (0.7%)	0.228

LOS: length of stay; DVT: deep venous thrombosis; PE: pulmonary embolism. Wound complications included exudation, bleeding, swelling, infection and impaired wound healing.

*p* value calculated using independent t-test, Pearson chi-square test or Fisher exact test.

## Discussion

Postoperative persistent pain following TKA is a collective concern for surgeons and patients [17]. Effective pain management after TKA could decrease complication risk, promote earlier rehabilitation, improve patient’s satisfaction and quality of life [18–20]. When conventional measures, such as NSAIDs and physical therapy, had poor efficacy, opioid drugs could be an available remedy in this study. Thus, the risk factor associated with opioid use could be responsible for the poor pain control after primary TKA. Because of the adverse effects of severe pain and opioid use, effective pain control and recognition of the risk factors for opioid use could guide provider and hospital system approach to managing postoperative pain after TKA [20, 21].

Substantial previous studies focused on the prevalence and prolonged opioid use instead of simple opioid use after TKA [10, 12, 13]. A retrospective cohort study performed by Robert et al indicated that younger age, preoperative NSAIDs and opioid use as well as some comorbidities were significant risk factors associated with prolonged postoperative opioid use after TKA [13]. In addition, Bedard and his colleagues determined that preoperative opioid use, a higher comorbidity score, rheumatoid arthritis, smoking and the benzodiazepine utilization were strong predictors for persistent opioid use postoperatively following TKA [10]. The research conducted by Kim et al reached a similar conclusion [12]. To our best knowledge, this study is the first one to determine the risk factors for the postoperative opioid use following primary TKA. Following an enhanced-recovery programme, the longer operative time is a risk factor while TXA use is a protective risk for opioid use after primary TKA.

It was reported that operative time, intraoperative blood loss and postoperative pain in TKA were in close positive correlation. Extended operative time could lead to more intraoperative blood loss, further resulting in increasing pain after TKA [22, 23]. Consequently, reducing operative time and blood loss was important step for postoperative pain care. Besides, smoking might also increase the risk for opioid use after TKA (*p* = 0.054). It was noted that smoking could cause more perioperative blood loss and higher complication risks [24]. Cryar et al demonstrated that preoperative smoking was the risk factor for narcotic use after TKA [25]. In addition, smoking was also a strong risk factor for persistent opioid use after primary TKA [12].

In this study, we found that application of TXA could reduce the risk of persistent pain and the risk of opioid use after TKA. As an antifibrinolytic agents, TXA could inhibit hyperfibrinolysis, reduce blood loss, alleviate joint swelling and relieve pain [26, 27]. Related studies also showed that patients could have less pain, less knee swelling and better knee function with the usage of multiple boluses of TXA following TKA [27, 28]. In addition to reducing blood loss, TXA have a synergistic effect to relieve pain after orthopaedic procedures. Currently, the application of TXA is an essential measure for blood management in enhanced recovery protocols.

The patients in opioid group sustained worse pain, longer LOS, higher incidence of nausea and vomiting after primary TKA. Opioid use was associated with increased LOS after TKA, which is consistent with previous studies [8, 12]. Halawi et al showed that opioid-based analgesia was significantly associated with increased LOS [8]. Another literature supported this result, showing that opioid use could lead to longer hospitalizations [11]. In addition, nausea and vomiting, which could increase complication risk and delay postoperative recovery, may be the side effects of opioid use [29]. So, it is necessary to take measures to prevent nausea and vomiting.

There are some limitations in this study. Firstly, we do not enroll preoperative medication history, such as sedative and opioid because related case data is incomplete. It was reported that preoperative benzodiazepine and opioid use was strong predictor for postoperative opioid use in TKA [10, 12]. Secondly, as a retrospective study, all data is from electronic medical record system and the level of evidence is still low. In addition, the follow-up time is only 30 days while chronic pain after TKA may continue more than 6 months [12]. So, the follow-up time in this study is insufficient to evaluate entire patients with opioid use after primary TKA. In a word, further studies with higher evidence level are requisite.

## Conclusion

Considering the adverse health effects of opioid use, strategies need to be developed to prevent persistent opioid use. Reducing operative time and the application of tranexamic acid could lower the risk of opioid use with an enhanced-recovery programme after primary TKA.

## Data Availability

The datasets used and/or analyzed during the current study are available from the corresponding author on reasonable request.

## Abbreviations

TKA: Total knee arthroplasty
LOS: Length of stay
OR: Odds ratio
CI: confidence interval
NSAIDs: Non-steroidal anti-inflammatory drugs
DVT: Deep venous thrombosis
PE: Pulmonary embolism
TXA: Tranexamic acid
VAS: Visual Analogue Scale
